# Assessment of factors related to individuals who were never treated during mass drug administration for lymphatic filariasis in Ambon City, Indonesia

**DOI:** 10.1371/journal.pntd.0010900

**Published:** 2022-11-11

**Authors:** Christiana Rialine Titaley, Caitlin M. Worrell, Iwan Ariawan, Yuniasih M. J. Taihuttu, Filda de Lima, Sazia F. Naz, Bertha J. Que, Alison Krentel

**Affiliations:** 1 Faculty of Medicine, Pattimura University, Poka Campus, Ambon, Indonesia; 2 Division of Parasitic Diseases and Malaria, US Centers for Disease Control and Prevention Atlanta, United States of America; 3 Swiss Tropical and Public Health Institute (Swiss TPH), Basel, Switzerland; 4 University of Basel, Basel, Switzerland; 5 Center for Health Research Universitas Indonesia, Faculty of Public Health Universitas Indonesia, UI campus, Depok, West Java, Indonesia; 6 Bruyère Research Institute, Ottawa, Canada; 7 School of Epidemiology and Public Health, University of Ottawa, Ottawa, Canada; Erasmus MC, NETHERLANDS

## Abstract

**Background:**

One challenge to achieving Lymphatic filariasis (LF) elimination is the persistent coverage-compliance gap during annual mass drug administration (MDA) and the risk of ongoing transmission among never treated individuals. Our analysis examined factors associated with individuals who were never treated during MDA.

**Methods:**

Data were derived from two cross-sectional surveys conducted in Waihaong and Air Salobar Health Center in 2018 and 2019. We analyzed information from 1915 respondents aged 18 years or above. The study outcome was individuals who self-reported never treatment during any round of MDA. All potential predictors were grouped into socio-demographic, health system, therapy and individual factors. Logistic regression analyses were used to examine factors associated with never treatment in any year of MDA.

**Results:**

Nearly half (42%) of respondents self-reported they were never treated during any round of MDA. Factors associated with increased odds of never treatment were respondents working in formal sectors (aOR = 1.75, *p = 0*.*040*), living in the catchment area of Waihaong Health Center (aOR = 2.33, *p = 0*.*029*), and those perceiving the possibility of adverse events after swallowing LF drugs (aOR = 2.86, *p<0*.*001*). Respondents reporting difficulty swallowing all the drugs (aOR = 3.12, *p<0*.*001*) and having difficulties remembering the time to swallow the drugs (aOR = 1.53, *p = 0*.*049*) also had an increased odds of never treatment. The highest odds of never treatment were associated with respondents reporting almost none of their family members took LF drugs (aOR = 3.93, *p<0*.*001*). Respondents confident that they knew how to swallow LF drugs had a reduced odds (aOR = 0.26, *p<0*.*001*) of never treatment.

**Conclusions:**

Efforts to reassure community members about adverse events, specific instructions on how to take LF drugs, and improving awareness that MDA participation is part of one’s contribution to promoting community health are essential drivers for uptake with LF drugs during MDA.

## Author summary

Lymphatic filariasis (LF) remains an important public health issues in endemic areas in Indonesia. One potential challenge to achieving LF elimination is the risk posed by never treatment during mass drug administration (MDA). Although Ambon City in Maluku Province, Indonesia, initiated its annual MDA in 2009, the microfilaria rate in 2016 still exceeded the threshold for elimination as a public health problem. In 2018 and 2019, we conducted a community-based study in the catchment areas of Air Salobar and Waihaong Health Centers, Ambon to improve treatment coverage and reduce the coverage-compliance gap in MDA for LF. Using data from the two cross-sectional surveys, our analysis examined factors associated with never treatment. We found the role of employment, healthcare catchment area, perceived possibility of side effects after swallowing LF drugs, difficulties in swallowing and remembering when to swallow all LF drugs and knowledge of how to swallow LF drugs were associated with the likelihood of never treatment. Recommendations to improve MDA uptake include the need to reassure community members about side effects, provide specific instructions on how to take LF drugs, and promote the message that MDA participation is part of one’s contribution to community health.

## Introduction

Lymphatic filariasis (LF) is a mosquito-borne disease that causes lymphoedema, elephantiasis, and hydrocele in individuals who have been infected, ultimately leading to permanent disability for many people [1]. An estimated 858.3 million people live in endemic areas where preventive chemotherapy is required to prevent and treat infection [2]. Since 2000, the Global Programme to Eliminate Lymphatic Filariasis (GPELF) has worked towards the goal of eliminating LF as a public health problem through two pillars: (1) the interruption of transmission with mass drug administration (MDA) and (2) morbidity management and disability prevention in people affected by LF disease [1].

Over the last twenty years, significant progress has been made, including delivering 8.6 billion treatments to 925 million people worldwide [2]. Seventeen countries have been certified as eliminating LF [2]. However, despite these many gains, efforts are still required to achieve LF elimination in many endemic countries [3,4]. One of the persistent challenges is identifying individuals who may have been missed during the MDA and, in particular, individuals who may have never been treated over the lifetime of the LF elimination program. In the literature, common terms used to describe these individuals include *systematic non-compliers*, *persistent non-compliers*, and *systematic non-adherence* [2,5–9]. These individuals have been defined by their self-reported lack of treatment during any MDA round [8]. In studies in Haiti [5] and Egypt [10], individuals who reported never treatment during any round of MDA were more likely to be positive for microfilaremia. The role of these individuals as potential reservoirs of infection in transmission failures or hotspots warrants further investigation.

As the global community approaches the next ten years of the GPELF and works towards achieving the World Health Organization’s NTD 2030 Roadmap [11], identifying and understanding never treatment during MDA becomes even more critical, especially in those countries where LF transmission persists. To date, there has been limited identification of never treatment in published MDA coverage assessments. Identifying the prevalence and distribution of never treatment will be necessary to discern how to reach these individuals and secure their participation in the program.

Indonesia is one of the largest LF-endemic countries in the global program. There are 236 districts/cities endemic for LF out of 514 districts/cities in the country [12]. Indonesia is also the only country globally with three species of filarial worms causing LF (*Wuchereria bancrofti*, *Brugia timori*, and *B*. *malayi*). Efforts to address LF in Indonesia began in 1975, particularly in highly endemic areas for LF [13]. In 2015, the microfilaria rate in Indonesia was 4.7%, a decrease from 19.5% in 1980 [12].

Some challenges to achieving elimination as a public health problem in Indonesia have been highlighted in previous studies. One challenge is the difficulty of achieving sufficient treatment coverage (>65%) in urban areas where there is high population mobility, diverse socio-demographic characteristics and education levels, and a lack of awareness of LF in the community [8,14,15]. Another challenge is the persistence of a coverage-compliance gap [16] in some areas whereby distributed drugs are not consumed, indicating the possibility of poor adherence to directly observed therapy [14].

As the Indonesian health system is decentralized, each endemic district must cover the required costs of the operations of the MDA using the donated medicines procured by the national LF program. Therefore, variability in the budgets provided for MDA at the district level can impact MDA delivery [8,14,17].

Ambon City in Maluku Province is an LF-endemic area in Indonesia that initiated its annual MDA using diethylcarbamazine citrate (DEC) and albendazole in 2009. Despite seven years of annual MDA, the microfilaria rate in Ambon in 2016 (9.4%) still exceeded the threshold, indicating the need for MDA [18]. After persistently low coverage (an average of 49%) in the first five years, in 2015, Ambon City was required to conduct a five additional annual rounds of MDA [18]. A study conducted in 2016 within the community living in the catchment area of two health centers in Ambon, i.e., Waihaong and Air Salobar Health Centers, found a substantial coverage-compliance gap in the populations served [19]. In Waihaong, among people receiving LF drugs, only 63.7% reported swallowing the received drugs; in Air Salobar, only 49.3% reported swallowing them. These findings indicated that present levels of non-compliance with MDA in Ambon City required urgent attention if LF elimination is to be achieved. This is further highlighted in the context of modeling studies that demonstrate the impact of persistent low coverage on the overall number of MDA rounds required to reach LF elimination [9,20].

In 2018 and 2019, the Faculty of Medicine, Pattimura University in Ambon, conducted a study with technical support from the U.S. Centers for Disease Control and Prevention (CDC) and the Bruyère Research Institute (Canada) to improve treatment coverage and reduce the coverage-compliance gap in MDA for LF through enhanced and tailored social mobilization messages. The study was carried out in the catchment areas of Air Salobar and Waihaong Health Centers. This manuscript describes a sub-analysis using data from two cross-sectional surveys, to examine factors associated with never treatment during any MDA rounds to better understand the prevalence and drivers of never treatment.

## Methods

### Ethics statement

This study received ethical approval from the Ethics Committee Faculty of Medicine Pattimura University (No. 112/FK-KOM.ETIK/VIII/2018). The Ministry of Health Republic of Indonesia, the Health Office of Maluku Province and Ambon City, Health Centers of Waihaong and Air Salobar, and all village administrators provided permission for this study. This activity was reviewed by CDC and was conducted consistent with applicable federal law and CDC policy [see e.g., 45 C.F.R. part 46, 21 C.F.R. part 56; 42 U.S.C. §241(d); 5 U.S.C. §552a; 44 U.S.C. §3501 et seq.]. The activity was approved by the Associate Director of Science at the U.S. Centers for Disease Control and Prevention (Tracking #2018–383). Written informed consent was obtained from all respondents interviewed in this study. No personal identifiers were collected, and the data were restricted to study personnel.

### Definitions

The use of the terms coverage and compliance vary across the literature. For this paper, the following definitions were used to distinguish potential coverage-compliance gaps in the MDA [16]. *Coverage* refers to individuals who reported receiving LF drugs during the most recent MDA. *Compliance* is defined as those individuals who reported receiving and swallowing LF drugs during the most recent MDA (also defined as drug coverage in some literature) [21,22]. The term *“never treatment”* is used to describe individuals who report never swallowing LF drugs during *any* MDA rounds. All variables are self-reported.

### Data sources and study area

Data analyzed in this study were derived from two cross-sectional surveys (baseline and endline) conducted in September 2018 and January 2019, respectively. The studies used data from a representative sample of the population eligible for MDA and the RANAS framework [23], which provides five domains (risks, attitudes, norms, abilities, and self-regulation) to understand coverage and compliance. To understand never treatment, we used results from a baseline study following the 2017 MDA, and an endline study following the 2018 MDA. In this paper, results from both surveys were combined. In the interval between the baseline and endline, the study team and the local health centers conducted a period of intervention. This intervention involved designing social mobilization strategies and messages to address the socio-behavioral factors that were associated with swallowing LF drugs based on the results of the baseline survey. Social mobilization strategies were implemented in two half-day briefing sessions for MDA distribution staff (cadres) and community/religious leaders. Some topics were discussed in those sessions, including the importance of intersectoral collaboration during the MDA, practical approaches that could be applied when distributing drugs and the use of some educational materials to promote compliance with swallowing LF drugs.

The study was carried out in the catchment areas of Waihaong and Air Salobar Health Centers in Ambon City, Indonesia. These areas were chosen as previous research demonstrated that Air Salobar Health Center health catchment area represented an area with adequate coverage yet poor reported compliance. In contrast, the population living in the catchment area of Waihaong Health Center represented an area with a higher yet still inadequate compliance [19].

Waihaong and Air Salobar Health Centers are two of 22 health centers in the administrative region of Ambon City. The Waihaong Health Center is a primarily coastal area encompassing 62.9 km^2^ that serves approximately 21,004 people from three villages, i.e., Waihaong, Silale, and Urimessing villages [24]. The catchment area of Air Salobar Health Center is 82.3 km^2^, covering two villages, i.e., Nusaniwe and Kudamati villages. The total population residing in Air Salobar Health Center in 2018 was 26,762 people [25].

### Study design and sampling of participants

The research team conducted an observational epidemiological study to investigate the socio-behavioral determinants of coverage and compliance in these two primary health care center catchment areas. The current subset analysis focused on associations of socio-behavioral risk factors of participants who reported never treatment during any round of MDA. We used information from all respondents involved in the study.

The sample size from each site in each survey was calculated to detect a mean difference in the mean behavioral score between compliers and non-compliers (mean difference of 1.0 points on a 5.0 point scale with a standard deviation of 2.5), assuming a (two-sided) α of 0.05 and a power of 0.80.

During the baseline survey, a two-stage cluster method was employed to select respondents. Twenty sub-hamlets were selected at each site, using the probability proportional to estimated size (PPS) method. The study field coordinators worked with local health center staff to create a list of sub-hamlets as the primary sampling unit. Within each sub-hamlet, enumerators obtained the list of all households in the sub-hamlet from the village administrator. Enumerators randomly selected 25 and 23 households per sub-hamlet in Air Salobar and Waihaong, respectively, and invited one randomly selected household resident to respond to the questionnaire. During the endline survey, identical methods were used; however, the sub-hamlets and households were re-randomized.

### Survey instruments and field personnel

After obtaining written informed consent, interviews were carried out in respondents’ houses, using a pre-tested and structured questionnaire. The questionnaire, written in Indonesia’s official language Bahasa Indonesia, gathered the individual’s key demographic data (i.e., age, sex, religion, education, occupation, income), knowledge of the MDA, coverage of LF drugs, compliance with swallowing LF drugs, history of compliance and behavioral factors that could influence compliance. The behavioral variables were adapted from the RANAS framework as developed by Mosler [23]. Data for both surveys were collected electronically using hand-held devices. The questionnaire was developed and administered on Dimagi’s CommCare application (Dimagi, Cambridge, MA) [26] for both surveys.

For both baseline and endline surveys, a team of enumerators was recruited for each site. Each team consisted of one field coordinator and nine enumerators. All field personnel attended a four-day training program conducted by researchers in the Faculty of Medicine, Pattimura University. The training included practice sessions in the surrounding area of the training site to standardize the interview techniques and ensure that all field workers understood each question in the questionnaire and the sampling methodology. All collected information was monitored regularly by a data manager in charge of all data submitted to the Commcare server.

### Variables

The study outcome variable was self-reported never treatment, constructed based on a question, "*how many times have you ever taken LF drugs*?" Participants were oriented by showing them the promotional materials from the most recent LF campaigns and photos of LF drugs. Respondents who responded "never or zero times" were categorized as “never treated,” otherwise, participants were considered as “ever treated” regardless of their participation in one or more than one MDA.

Our analysis considered 33 potential predictors of never treatment in the previous rounds of MDA for LF. To examine the factors associated with never treatment in the two study sites, we adapted the WHO framework of four dimensions of adherence [27]. The potential predictors are related to four dimensions: (1) socio-economic factors, (2) health system-related factors, (3) therapy-related factors, and (4) individual-related factors. The description of therapy and individualrelated variables is shown in S1 Table.

In the respondents’ occupation variable, we classified government/private employee/military/police officers as formal sector workers. The income variable included in the socio-demographic factors was based on the monthly regionally-mandated minimum wages of Ambon City (~USD154). The regional minimum wage in Indonesia is determined by the *Ministry* of *Manpower* and Transmigration, Republic of Indonesia, and is commonly known amongst the population. This variable was used in earlier studies in Indonesia [14,15].

One of the variables used in the individual-related factors was respondents’ knowledge and awareness of LF and MDA. This variable was constructed based on five questions: (a) ever heard of the MDA; (b) perceived susceptibility to contracting LF; (c) knowledge that LF is not a hereditary disease; (d) knowledge of preventive measures of LF; and (e) perceived personal obligation to swallow LF drugs. For each question, a score of "1" (one)" was assigned to those responding with correct factual knowledge or awareness, while "0" (zero) was assigned for answers that were incorrect factual knowledge or low awareness. Respondents were classified into a high and low level of knowledge and awareness based on the distribution of effect size (odds ratio) for never treatment obtained from the bivariate logistic regression analysis. We used the cut-off where the odds of never treatment in the logistic regression changed considerably [28].

### Data analysis

**Fig 1:**
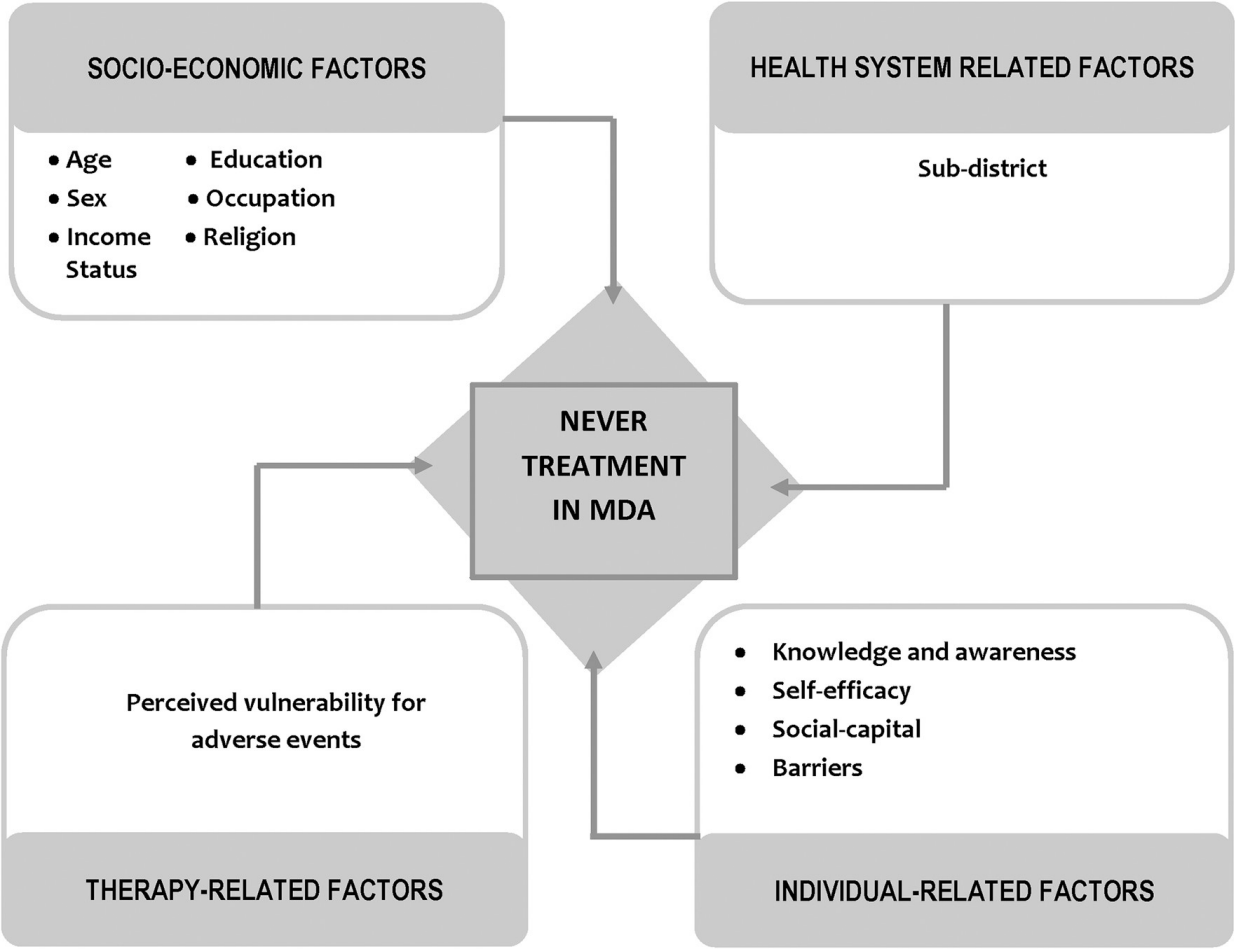
Analytical framework used to analyze factors associated with “never treated” among survey respondents in the catchment area of Waihaong and Air Salobar Health Center, Ambon City.

In our analysis, contingency tables were used to examine the frequency distribution of all variables. We identified 33 variables from the dataset potentially related to never treatment during MDA. The least absolute shrinkage and selection operator (lasso) was then performed to select predictors included in the regression model [29]. From the 33 variables, only nine variables were selected. Those variables were: (1) Perceived vulnerability for adverse events, (2) Knowledge and awareness of LF and MDA, (3) Receiving LF drugs despite efforts, (4) Level of difficulty swallowing LF drugs, (5) Level of difficulty remembering to swallow LF drugs, (6) Perceived family participation, (7) Perceived level of support from important people to swallow LF drugs, (8) Action knowledge instructions on LF drugs (level of confidence in the instructions on how to take LF drugs (e.g., dose and timing), and (9) Level of confidence in the ability to swallow LF drugs several years in a row. All of these variables were included in the logistic regression model in addition to all socio-demographic variables (eight variables), even if not selected by the lasso model (Fig 1).

Univariable logistic regression analyses were used to examine the relationship between each potential predictor and our outcome variable. The estimated measures of the association were assessed using the odds ratio (OR). This was followed by multivariable logistic regression analyses to assess the relationship between each potential predictor and our outcome variable after adjusting for all other covariates, using the adjusted odds ratio (aOR). A significance level of 0.05 was used. Stata/MP software (version 13.1; StataCorp LLC, College Station, TX) was used for all analyses. The syv command in Stata was used to take into account the sampling probability used in the survey.

## Results

**Fig 2:**
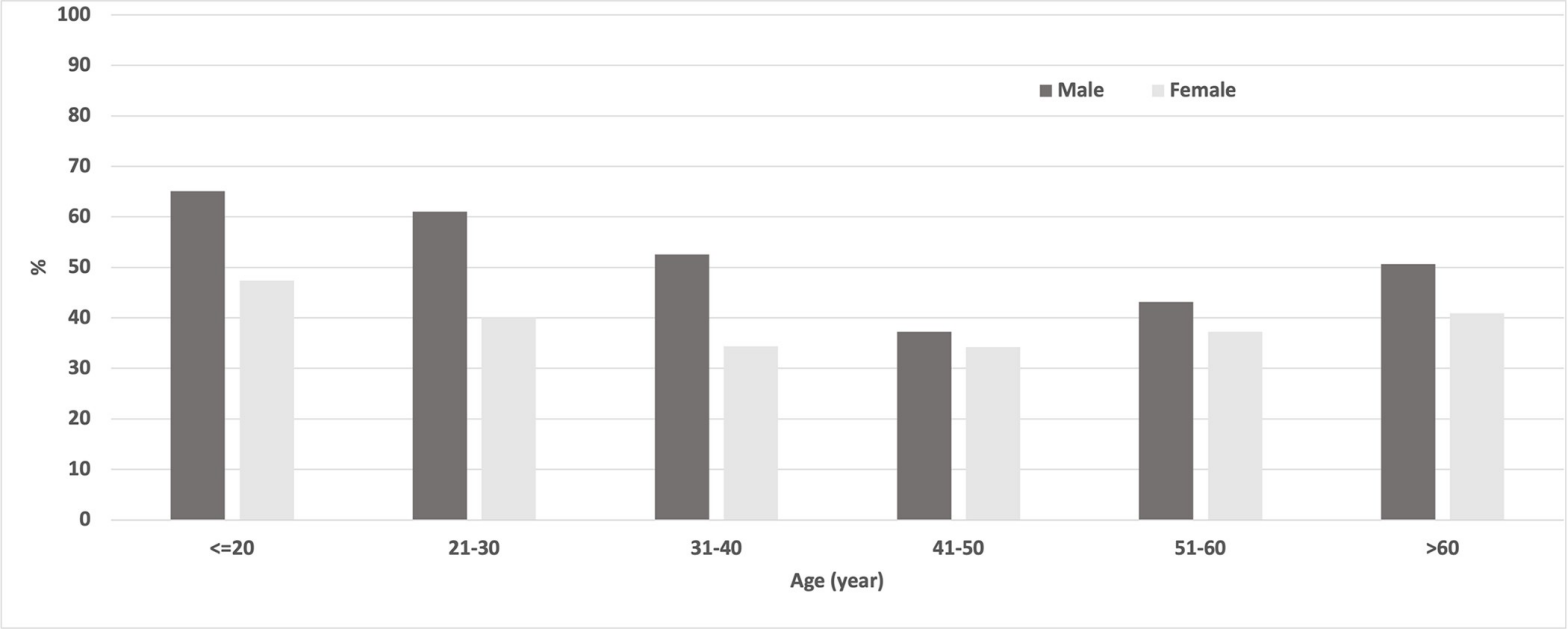
Frequency distribution of never treated by respondents’ age group and sex in the catchment area of Waihaong and Air Salobar Health Center, Ambon City.

Our study analyzed information collected from 1915 respondents (951 from the baseline and 964 from the endline surveys). The frequency distribution of variables included in this analysis is shown in Table 1. There were significant differences in some socio-demographic factors among respondents participating in the baseline vs. endline surveys (respondents’ level of education, sex, and occupation). At endline, we also found significantly higher proportions of positive responses than the baseline, except for the variable of the level of confidence in receiving LF drugs despite effort. Fig 2 shows the frequency distribution of never treatment by respondents’ age group and sex. A high percentage of never treatment was found in the young age groups, reduced in the middle age group while increasing again in the older age group. There was also a consistently lower percentage of never treatment across females than males across all age groups. The frequency distribution of variables not included in the logistic regression model by the time of the survey and respondents’ compliance status is in S2 Table.

**Table 1:** Frequency distribution of factors analyzed in this study by time of survey and compliance.

Variable	Time of survey		*p*	Total baseline and endline (n)	% of total baseline and endline	Never treated (%) of total baseline and endline
	Baseline (n = 951)	Endline (n = 964)				
**Systematic compliance**			*<0*.*001*			
Ever treated (= 0)	45.43	70.85		1115	58.22	
Never treated (= 1)	54.57	29.15		800	41.78	
**Age**			*0*.*053*			
≤ 20 years old	4.52	5.91		100	5.22	55.00
21–30 years old	21.03	19.81		391	20.42	47.57
31–40 years old	22.29	23.24		436	22.77	40.14
41–50 years old	24.29	19.71		421	21.98	35.15
51–60 years old	15.14	18.67		324	16.92	39.20
>60 years old	12.20	12.66		238	12.43	44.12
**Education**			*<0*.*001*			
No schooling	8.10	0.83		85	4.44	48.24
Primary School	7.47	6.74		136	0.71	38.24
Junior High School	8.52	10.58		183	9.56	39.34
Senior High School	50.05	55.50		1011	52.79	40.16
University/Academy	25.87	26.35		500	26.11	45.80
**Sex**			*0*.*003*			
Male	30.18	36.93		643	33.58	50.39
Female	69.82	63.07		1272	66.42	37.42
**Occupation**			*0*.*047*			
Housewife	37.64	36.83		713	37.23	34.50
Formal sector workers	13.67	19.50		318	16.61	49.06
Trader/entrepreneur	19.03	16.80		343	17.91	37.32
Farmer/fisherman/laborer	8.83	7.47		156	8.15	48.72
Student	7.05	7.57		140	7.31	55.00
Retired person	12.30	9.96		213	11.12	48.36
**Income status**			*0*.*050*			
Lower than the monthly minimum wages	40.48	38.90		760	39.69	38.03
Equals to the monthly minimum wages	16.82	23.65		388	20.26	39.69
Higher than the monthly mißnimum wages	42.69	37.45		767	40.05	46.54
**Religion**			*0*.*820*			
Muslim	47.84	49.59		933	48.72	41.26
Christian (Protestant / Catholic)	51.95	50.31		979	51.12	42.29
**Health system-related factors**						
Sub-district			*0*.*950*			
Air Salobar	50.37	49.90		960	50.13	39.17
Waihaong	49.63	50.10		955	49.87	44.40
**Therapy-related factors**						
**Perceived vulnerability for adverse events**			*<0*.*001*			
Impossible/small	46.16	41.39		838	43.76	26.01
Neutral	25.24	37.24		599	31.28	55.09
Possible/very possible	28.60	21.37		478	24.96	52.72
**Individual-related factors**						
**Knowledge and awareness of LF and MDA**			*0*.*011*			
Low level	14.20	8.92		221	11.54	73.30
High level	85.80	91.08		1694	88.46	37.66
**Perceived difficulty in receiving LF drugs**			*0*.*112*			
Easy	77.50	78.53		1494	78.02	34.27
Neutral	10.52	12.66		222	11.59	63.51
Difficult	11.99	8.82		199	10.39	73.87
**Level of difficulty in swallowing all LF drugs**			*0*.*003*			
Easy	63.41	71.37		1291	67.42	25.33
Neutral	12.72	14.94		265	13.84	68.68
Difficult	23.87	13.69		359	18.75	81.06
**Remember swallow LF drugs**			*0*.*005*			
Easy	54.47	50.10		1001	52.27	25.37
Neutral	24.29	35.06		569	29.71	55.01
Difficult	21.24	14.83		345	18.02	67.54
**Perceived family participation**			*<0*.*001*			
Don’t know	6.10	5.60		112	5.85	63.39
Almost none	38.91	22.41		586	30.60	87.20
Less than half	4.00	6.33		99	5.17	50.51
Half	11.25	14.73		249	13.00	34.54
More than half	2.73	10.79		130	6.79	26.92
Almost all	37.01	40.15		739	38.59	6.36
**Support from important people in receiving LF drugs**			*<0*.*001*			
Neutral/unsupportive	35.86	18.46		519	27.10	69.94
Supportive	64.14	81.54		1396	72.90	31.30
**Action knowledge instructions on LF drugs**			*0*.*015*			
Not sure	42.38	34.85		739	38.59	72.80
Sure	57.62	65.15		1176	61.41	22.28
**Swallowing LF drugs over time**			*0*.*002*			
Not sure	41.75	32.47		710	37.08	68.59
Sure	58.25	67.53		1205	62.92	25.98

**Fig 3:**
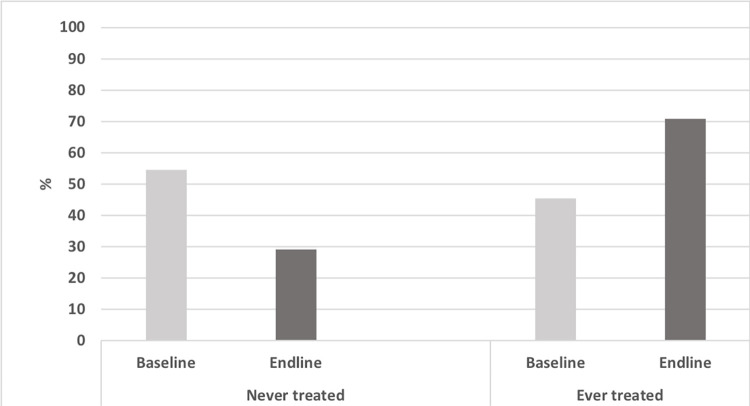
Proportion of never treated in the catchment area of Waihaong and Air Salobar Health Center, Ambon City at the the time of surveys. Note: p-value refers to the difference between baseline and endline forever treatment (chi-square).

In total, 41.78% (95% Confidence Interval (CI): 38.27–45.36) of respondents reported never treatment during any round of MDA. Significantly more people reported never treatment in the baseline survey (54.57%, 95% CI: 50.38%-58.7%) than in the endline survey (29.15%, 95%CI: 25.68%-32.88%) (Fig 3). Actions between the baseline and endline surveys by primary health care centers to address some of the coverage challenges in the two health centers might have accounted for this difference (e.g., reduction of never treatment).

The frequency distribution of all variables included in this analysis by compliance status is also presented in Table 1. A higher percentage of never treatment during MDA for LF was found in the catchment area of Waihaong than Air Salobar Health Center. Respondents who reported barriers to treatment, such as the difficulty of swallowing LF drugs, were more likely to be never treated than those reporting no barriers to treatment. Similarly, those who did not receive social support from their family or surroundings were more likely to be never treated than those receiving support.

Table 2 shows factors associated with never treatment during MDA for LF. A significant reduction of the odds for never treatment was found in the endline survey (aOR = 0.18, *p<0*.*001*). Those working in the formal sector had an increased likelihood of never treatment than housewives (aOR = 1.75, *p = 0*.*040*). Amongst health system-related factors, respondents living in the catchment area of Waihaong Health Center had an increased odds of never treatment compared to those living in the catchment area of Air Salobar Health Center (aOR = 2.33, *p = 0*.*029*). In the therapy-related factors, respondents who perceived some vulnerability to developing adverse events following taking LF drugs had an increased odds of never treatment compared with those who perceived a low possibility of developing adverse events (aOR = 2.86, *p<0*.*001*).

**Table 2:** Univariate and multivariate analyses for factors associated with never treated in during MDA for Lymphatic Filariasis in Waihaong and Air Salobar Air Center, Kota Ambon.

Variable	Univariate				Multivariate			
	OR	95% CI		*p*	aOR	95% CI		*p*
**Period of survey**								
Baseline	1.00				1.00			
Endline	0.34	0.27	0.43	*<0*.*001*	0.18	0.12	0.27	*<0*.*001*
**Socio-demographic factors**								
**Age**								
≤20 years old	1.00				1.00			
21–30 years old	0.74	0.50	1.11	*0*.*140*	1.00	0.40	2.47	*0*.*999*
31–40 years old	0.55	0.37	0.82	*0*.*004*	1.00	0.41	2.41	*0*.*997*
41–50 years old	0.44	0.31	0.63	*<0*.*001*	0.71	0.28	1.80	*0*.*462*
51–60 years old	0.53	0.35	0.79	*0*.*003*	0.91	0.32	2.55	*0*.*851*
>60 years old	0.65	0.43	0.96	*0*.*032*	1.10	0.46	2.65	*0*.*828*
**Education**								
No schooling	1.00				1.00			
Primary School	0.66	0.37	1.19	*0*.*167*	0.66	0.24	1.82	*0*.*415*
Junior High School	0.70	0.45	1.09	*0*.*108*	1.21	0.58	2.49	*0*.*607*
Senior High School	0.72	0.50	1.05	*0*.*084*	1.00	0.49	2.05	*0*.*998*
University/Academy	0.91	0.61	1.34	*0*.*617*	0.93	0.43	2.01	*0*.*848*
**Sex**								
Male	1.00				1.00			
Female	0.59	0.50	0.70	*<0*.*001*	1.06	0.76	1.49	*0*.*719*
**Occupation**								
Housewife	1.00				1.00			
Formal sector workers	1.83	1.41	2.37	*<0*.*001*	1.75	1.02	3.03	*0*.*044*
Trader/entrepreneur	1.13	0.86	1.49	*0*.*378*	1.00	0.56	1.81	*0*.*989*
Farmer/fisherman/labor	1.80	1.22	2.66	*0*.*004*	1.66	0.90	3.07	*0*.*103*
Students	2.32	1.51	3.57	*<0*.*001*	1.57	0.61	4.01	*0*.*341*
Retired person	1.78	1.29	2.45	*0*.*001*	1.24	0.67	2.29	*0*.*484*
**Income status**								
Lower than the monthly minimum wages	1.00				1.00			
Equals to the monthly minimum wages	1.07	0.82	1.40	*0*.*596*	0.91	0.62	1.35	*0*.*636*
Higher than the monthly minimum wages	1.42	1.14	1.76	*0*.*002*	1.08	0.72	1.64	*0*.*696*
**Religion**								
Muslim	1.00				1.00			
Christian (Protestant / Catholic)	1.04	0.80	1.36	*0*.*755*	1.82	0.91	3.64	*0*.*087*
**Healthy system-related factors**								
Sub-district								
Air Salobar	1.00				1.00			
Waihaong	1.24	0.93	1.65	*0*.*136*	2.33	1.10	4.94	*0*.*029*
**Therapy-related factors**								
**Perceived vulnerability for adverse events**								
Impossible/small	1.00				1.00			
Neutral	3.49	2.68	4.55	*<0*.*001*	2.90	1.97	4.27	*<0*.*001*
Possible/very possible	3.17	2.45	4.10	*<0*.*001*	2.86	1.99	4.12	*<0*.*001*
**Individual-related factors**								
**Knowledge and awareness of LF and MDA**								
Low level	1.00				1.00			
High level	0.22	0.16	0.31	*<0*.*001*	0.96	0.58	1.57	*0*.*861*
**Perceived difficulty in receiving LF drugs**								
Easy	1.00				1.00			
Neutral	3.34	2.42	4.60	*<0*.*001*	0.94	0.55	1.60	*0*.*817*
Difficult	5.42	3.83	7.67	*<0*.*001*	1.47	0.90	2.38	*0*.*119*
**Level of difficulty in swallowing all LF drugs**								
Easy	1.00				1.00			
Neutral	6.46	4.60	9.09	*<0*.*001*	2.60	1.59	4.25	*<0*.*001*
Difficult	12.62	8.09	19.68	*<0*.*001*	3.12	1.84	5.31	*<0*.*001*
**Remember swallow LF drugs**								
Easy	1.00				1.00			
Neutral	3.60	2.73	4.73	*<0*.*001*	1.46	0.97	2.20	*0*.*067*
Difficult	6.12	4.18	8.96	*<0*.*001*	1.53	1.00	2.33	*0*.*049*
**Perceived family participation**								
Don’t know	1.00				1.00			
Almost none	3.93	2.39	6.49	*<0*.*001*	3.93	2.27	6.82	*<0*.*001*
Less than half	0.59	0.28	1.22	*0*.*152*	1.11	0.54	2.25	*0*.*777*
Half	0.30	0.17	0.54	*<0*.*001*	0.46	0.24	0.89	*0*.*022*
More than half	0.21	0.12	0.39	*<0*.*001*	0.91	0.49	1.68	*0*.*754*
Almost all	0.04	0.02	0.06	*<0*.*001*	0.11	0.06	0.19	*<0*.*001*
**Support from important people in receiving LF drugs**								
Neutral/unsupportive	1.00				1.00			
Supportive	0.20	0.16	0.25	*<0*.*001*	1.02	0.68	1.53	*0*.*917*
**Action knowledge instructions on LF drugs**								
Not sure	1.00				1.00			
Sure	0.11	0.08	0.14	*<0*.*001*	0.26	0.19	0.35	*<0*.*001*
**Swallowing LF drugs over time**								
Not sure	1.00				1.00			
Sure	0.16	0.12	0.22	*<0*.*001*	0.67	0.45	1.00	*0*.*051*

Of the individual-related factors, two barriers emerged as significant predictors: difficulty swallowing all LF drugs and difficulty remembering the time to swallow LF drugs. Respondents who were neutral (aOR = 2.60, *p<0*.*001*) or reported a high degree of difficulty in swallowing all LF drugs (aOR = 3.12, *p<0*.*001*) had an increased likelihood of never treatment during MDA compared with those who reported that it was easy swallowing LF drugs. Similarly, respondents who had difficulties remembering the time to swallow LF drugs also had an increased odds of never treatment (aOR = 1.53, *p = 0*.*049*).

Our analysis found a strong association between family support and respondents’ decisions to take LF drugs. Among the variables we assessed, the highest odds for never treatment during the MDA was associated with respondents reporting that almost none of their family members took LF drugs (aOR = 3.93, *p<0*.*001*). Similarly, we found low odds for never treatment among those reporting that almost all of the family took LF drugs (OR = 0.11, *p<0*.*001*).

For self-efficacy variables, respondents who were confident that they knew how to swallow LF drugs had significantly reduced odds (aOR = 0.26, *p<0*.*001*) for never treatment. We also found that respondents who were confident that they could swallow LF drugs for several years in a row had a lower odds of never treatment in the MDA than those who were not confident in doing so (aOR = 0.67, *p = 0*.*051*), with a borderline significance.

## Discussion

This study provides insights into the proportion of the population reporting never treatment in the catchment area of two primary health care centers in Ambon City, Indonesia. Nearly half of all study respondents self-reported never treatment during any round of MDA. There was a notable reduction in levels of individuals reporting never treatment between respondents in endline assessment compared with respondents in the baseline assessment, suggesting that interventions might have been successful in reaching some individuals for the first time in the subsequent MDA round. Of the socio-demographic indicators, only one occupation group (formal sector workers) had a strong relationship with never treatment in univariable/multivariable analyses. In terms of the catchment area of the primary health care centers, the context was different, including higher levels of never treatment in Waihong Health Center as compared to Air Salobar Health Center.

Individual perceptions, particularly fear, about side effects have been noted consistently in the literature as an essential barrier to LF MDA participation [7,8,21,22,30,31], even though the side effects seem to occur at fairly low levels in the population as demonstrated in a community-based safety study for LF MDA, and are mild and self-limited when they do occur [32]. In the Ambon data, the perception that side effects were possible after swallowing LF drugs during MDA was associated with never treatment. This suggested that this perception might have played a role in individuals’ decisions not to take the treatment during MDA over multiple rounds. Fear of side effects might be related to community norms or rumors circulating about the treatment or to a lack of care provided if side effects do occur [33]. Having a system in place to reassure community members about what to do in the event of side effects may minimize their importance amongst community members and reduce the fear associated with side effects and increase trust in the program [31,33].

Self-efficacy is a concept that encompasses people’s beliefs in their abilities to influence the events that affect their lives [34]. Within the context of MDA participation, self-efficacy has not been widely explored. Health service personnel and volunteers (or cadres) bring LF drugs directly to the community with the assumption that community members will know what to do when given the drugs, e.g., which drugs to swallow and which to chew. This study suggests that self-efficacy can play an important role in a person’s decision to take LF treatment during MDA. When individuals feel unsure about the instructions for swallowing LF drugs, they appear more likely to opt out of treatment. Currently, messaging for MDA does not often include information on how to swallow the drugs or the ability to chew the drugs, in the case of albendazole. The assumption that people will know how to take LF drugs might have led specific individuals to refrain from participating in MDA treatment in this study. This was highlighted in earlier research in Indonesia where respondents indicated a variety of ways they took the MDA treatment (e.g., as all at once, over the course of one day or over one week). It is our understanding that information about how to take drugs is not widely promoted in the social mobilization and messaging around MDA.

Although there are multiple definitions of social capital [35], for this research, we prefer Putman’s definition [36], which defines social capital as "features of social organization such as networks, norms, and social trust that facilitate coordination and cooperation for mutual benefit." Within the context of Indonesia, many communities have a history of cooperation for mutual benefit, with there are well-known examples of *gotong royong*, a practice where community members come together for a common goal (e.g., clearing a football field of long grass, cleaning a dirty area of the village, repairing the roof of a religious building). According to social norms, individual action for the benefit of the wider community or other household members has been shown in earlier studies to be associated with greater compliance [14,30,37]. In this study, we found that in Ambon, the norm of MDA participation within the household appears to be associated with an individual household member’s participation. There was an association between the respondent not taking the MDA treatment and knowing that no other household members took the MDA treatment. These results suggest that household clustering may exist for never treatment and related knowledge and awareness factors. This can have important implications for improving MDA following low coverage and/or evidence of never treatment. Identifying geographic areas where households may have been missed could help with reaching individuals for the first time in subsequent rounds as well as training drug distributors to ask if people in the household had ever taken MDA treatment before.

Finally, this research suggests the benefits of monitoring “never treatment” in addition to routine MDA coverage measurements. Previous Indonesian studies in Agam District and Depok City demonstrated the use of never treatment as one of the barometers of success of interventions implemented to increase coverage and compliance [14,38]. In this study, a reduction in levels of never treatment was detected between the baseline and endline data collection following a period of intervention between the MDA rounds. Further assessment of the use of the never treatment indicator to measure improvements to MDA is recommended in areas where higher levels are known or expected. It should be combined with existing measurements of coverage in use.

### Limitations

Some limitations in the study are worth noting. The baseline survey was carried out six months after the last MDA (2017), while the endline survey was conducted one month following the 2018 MDA, which may lead to differing levels of recall between the baseline and endline surveys. It was not possible to validate respondents’ coverage and compliance information, and the interviews were based solely on respondents’ recollections and self-reports. Although there may be recall bias, a study looking at recall in MDA coverage surveys found less recall bias than expected [39]. Furthermore, there might be other potential predictors for never treatment during the MDA that we did not include in our analysis as they were in the dataset, such as the length of time respondents lived in the study sites. However, this study included only respondents who had lived in the study sites since the 2017 MDA.

Additionally, the study team was not able to assess the impact of the study itself on the improvement of coverage in the study areas. The involvement and engagement of the research team in both health center catchment areas may have positively impacted the subsequent MDA. Exploring this issue is inherently challenging to draw conclusive reasons for never treatment due to the complexity and mutability of risk factors over time. The study design we used did not allow us to draw causal links between factors analyzed and never treatment, and we cannot rule out the possibility of reverse causality of the outcome and some associations. Many of the predictor variables could change over time, such as respondent’ current self-efficacy or knowledge, while never treatment is assessed over a long period of time in the past. So experience taking MDA could also increase a person’s self-efficacy. As a consequence, it is also possible that respondents’ participation in MDA over the years influenced the predictor variables.

## Conclusion

In summary, our analysis showed that efforts to improve coverage and compliance should consider never treatment. Efforts to reassure community members about side effects are important to promote uptake during an LF MDA. The health sector’s role, including drug deliverers, to convey specific messages to the community, such as instructions on how to take the drugs, are essential additions to social mobilization. These and better messaging around MDA could improve uptake in the never treated. As demonstrated here and in previous research in Indonesia, raising awareness in the community that participates in the MDA is part of one’s contribution to the health benefits of the household, and the community is an important consideration for MDA uptake in Ambon [15].

## Supporting information

S1 Table Description of analysis variables (therapy and individual-related factors). (DOCX)

S2 Table Frequency distribution of variables not included in the Univariate and Multivariate regression model on factors associated with “never treated” during MDA for Lymphatic Filariasis in Ambon City. (DOCX)
