# Supplementary material for: Assessment of factors related to individuals who were never treated during mass drug administration for lymphatic filariasis in Ambon City, Indonesia
Source: PLoS Negl Trop Dis. 2022 Nov 11;16(11):e0010900. doi: 10.1371/journal.pntd.0010900 (PMC9683609; doi:10.1371/journal.pntd.0010900)
Supplement: S1 Table — (DOCX) [file pntd.0010900.s001.docx]

**Supplementary Table 1.** Description of analysis variables (therapy and individual-related factors)

| **Category** | **Variable** | **Description** | **Question** |
| --- | --- | --- | --- |
| **Therapy-related factors** | | | |
|  | Perceived vulnerability for adverse events | Perceived vulnerability to develop adverse events following treatment | How likely do you think you are to have an adverse reaction to medication if you swallow LF drugs? |
| **Individual-related factors** | | | |
| **Knowledge and awareness** | Scaled variable with 12 elements | Scaled variable assessing awareness and knowledge about LF based on: 1) ever heard of MDA for LF; 2) perceived likelihood of developing lymphedema if not treated; 3) knowledge of correct prevention strategies for filariasis (8 elements); 4) knowledge that LF is not hereditary; 5) perceived personal obligation to swallow LF drugs | - |
| **Self-Efficacy** | Example for others | Perception of the extent to which taking LF drugs sets a good example for others | How strongly do you think that you give a good example to your children and/or your family if you swallow LF drugs? |
|  | Action knowledge instructions on LF drugs | Level of confidence in the instructions on how to take LF drugs (e.g., dose, timing, etc.) | Please describe your level of confidence that you have instructions on how to take LF drugs (e.g. dose, timing)? |
|  | Action knowledge AE help | Level of confidence in ability to receive help for adverse events (action knowledge) | Please describe your level of confidence that you know where to receive help if you have an adverse reaction? |
|  | Receiving despite effort | Level of confidence in receiving LF drugs despite effort (maintenance self-efficacy) | Please describe your level of confidence that you will be available to RECEIVE the LF drugs even if takes an effort to do so? |
|  | Level of difficulty (swallowing LF drugs) | Level of difficulty swallowing LF drugs | Please describe your level of confidence that you will be able to SWALLOW the LF even if takes an effort to do so? |
|  | Receiving LF drugs over time | Level of confidence in ability to receive drugs several years in a row (maintenance self-efficacy) | How confident are you that you will be available to RECEIVE LF drugs for several years in a row? |
|  | Swallowing LF drugs over time | Level of confidence in ability to swallow drugs several years in a row (maintenance self-efficacy) | How confident are you that you will be able to SWALLOW LF drugs for several years in a row? |
|  | Re-start taking LF drugs if missed | Level of confidence in ability to start taking drugs again after missing a year (recovery self-efficacy) | Imagine that you did not swallow the LF drugs one year. How confident are you that you could take them again the next year? |
|  | Finding drugs in DD absence | Level of confidence in ability to find the drugs if the drug distributor passed by while absent (recovery self-efficacy) | Imagine that you were not at home when the CDD passed by to deliver LF drugs. How confident are you that you would know where to find the drugs? |
| **Social-Capital** | Perceived community participation | Perceived proportion of other community members who swallowed LF drugs | What do you think, how many people in your community swallow LF drugs during the filariasis mass drug administration? |
|  | Perceived family participation | Perceived proportion of other family members who swallowed LF drugs | What do you think, how many people in your family swallow LF drugs during the filariasis mass drug administration? |
|  | Perceived promotion leaders | Perceived promotion to participate in the MDA from community leaders | Among leaders in your community, how much do they approve or should not swallow LF drugs? |
|  | Support from important people (receive LF drugs) | Perceived level of support from important people around you to receive LF drugs | How much do people important to you (e.g. head of household, friends, etc.) support you in RECEIVING LF drugs ? |
|  | Support from important people (swallow LF drugs) | Perceived level of support from important people around you to swallow LF drugs | How much do people important to you (e.g. head of household, friends, etc.) support you in SWALLOWING LF drugs? |
| **Barriers** | Effort to receive | Perceived level of effort required to receive LF drugs | Do you think you need to make a big effort to RECEIVE LF drugs? |
|  | Effort to swallow | Perceived level of effort required to swallow LF drugs | Do you think you need to make a big effort to SWALLOW LF drugs? |
|  | Trust in DD team | Level of trust in drug distribution team (instrumental beliefs) | Can you describe the level of confidence (trust) you had in the drug distribution team that gave you LF drugs? |
|  | Costs to receive | Perceived costs of receiving LF drugs | Which face comes closest to how you feel about the costs (e.g. transportation or missed time at school/work) associated with RECEIVING LF drugs? |
|  | Costs to swallow | Perceived costs of swallowing LF drugs | Which face comes closest to how you feel about the costs (e.g. transportation or missed time at school/work) associated with SWALLOWING LF drugs? |
|  | Level of difficulty (receiving LF drugs) | Level of difficulty of receiving LF drugs | Please describe your level of difficulty to RECEIVE LF drugs? |
|  | Level of difficulty (swallowing at once) | Level of difficulty swallowing all LF drugs at the same time | Please describe your level of difficulty SWALLOW all of LF drugs on the same day? |
|  | Remember receive LF drugs | Level of difficulty remembering to receive LF drugs | How difficult is it to remember to be available to RECEIVE LF drugs? |
|  | Remember swallow LF drugs | Level of difficulty remembering to swallow LF drugs | How difficult is it to remember to be to SWALLOW LF drugs? |
