# Supplementary material for: Assessment of factors related to individuals who were never treated during mass drug administration for lymphatic filariasis in Ambon City, Indonesia
Source: PLoS Negl Trop Dis. 2022 Nov 11;16(11):e0010900. doi: 10.1371/journal.pntd.0010900 (PMC9683609; doi:10.1371/journal.pntd.0010900)
Supplement: S2 Table — (DOCX) [file pntd.0010900.s002.docx]

**Supplementary Table 2.** Frequency distribution of variables not included in the Univariate and Multivariate regression model on factors associated with “never treated” during MDA for Lymphatic Filariasis in Ambon City

| **Variable** | **Time of survey** | | **Total baseline and endline (n)** | **% of total baseline and endline** | **Never treated**  **(%) of total baseline and endline** |
| --- | --- | --- | --- | --- | --- |
|  | **Baseline**  **(n=951)** | **Endline**  **(n=964)** |  |  |  |
| **Knowledge and awareness** |  |  |  |  |  |
| **Ever heard of MDA for LF** |  |  |  |  |  |
| Never | 0.00 | 8.61 | 83 | 4.33 | 77.11 |
| Ever | 100.00 | 91.39 | 1832 | 95.67 | 40.17 |
| **Perceived likelihood of developing lymphedema if not treated** | |  |  |  |  |
| Impossible | 64.98 | 57.57 | 1173 | 61.25 | 47.83 |
| Possible | 35.02 | 42.43 | 742 | 38.75 | 32.21 |
| **Knowledge that LF is not hereditary** |  |  |  |  |  |
| Hereditary | 21.35 | 14.94 | 347 | 18.12 | 51.59 |
| Not hereditary | 78.65 | 85.06 | 1568 | 81.88 | 39.60 |
| **Knowledge of correct prevention strategies for LF** |  |  |  |  |  |
| Low level | 65.51 | 70.23 | 1300 | 67.89 | 42.77 |
| High level | 34.49 | 29.77 | 615 | 32.11 | 39.67 |
| **Perceived obligation to take LF drugs** |  |  |  |  |  |
| Not compulsory | 12.83 | 2.59 | 147 | 7.68 | 85.03 |
| Low level | 32.70 | 31.85 | 618 | 32.27 | 54.69 |
| High level | 54.47 | 65.56 | 1150 | 60.05 | 29.30 |
| **Self-efficacy** |  |  |  |  |  |
| **Example for others** |  |  |  |  |  |
| Not sure | 25.97 | 33.61 | 571 | 29.82 | 56.92 |
| Sure | 74.03 | 66.39 | 1344 | 70.18 | 35.34 |
| **Action knowledge adverse events help** | |  |  |  |  |
| Not sure | 10.83 | 19.29 | 289 | 15.09 | 57.44 |
| Sure | 89.17 | 80.71 | 1626 | 84.91 | 38.99 |
| **Receiving despite effort** |  |  |  |  |  |
| Not sure | 36.07 | 38.80 | 717 | 37.44 | 56.76 |
| Sure | 63.93 | 61.20 | 1198 | 62.56 | 32.80 |
| **Level of difficulty (swallowing LF drugs)** |  |  |  |  |  |
| Not sure | 36.49 | 40.56 | 738 | 38.54 | 60.43 |
| Sure | 63.51 | 59.44 | 1177 | 61.46 | 30.08 |
| **Receiving LF drugs over time** |  |  |  |  |  |
| Not sure | 43.95 | 26.87 | 677 | 35.35 | 60.12 |
| Sure | 56.05 | 73.13 | 1238 | 64.65 | 31.74 |
| **Re-start taking LF drugs if missed** |  |  |  |  |  |
| Not sure | 43.11 | 28.53 | 685 | 35.77 | 67.30 |
| Sure | 56.89 | 71.47 | 1230 | 64.23 | 27.56 |
| **Finding LF drugs in drugs distributors absence** |  |  |  |  |  |
| Not sure | 23.76 | 27.07 | 487 | 25.43 | 61.19 |
| Sure | 76.24 | 72.93 | 1428 | 74.57 | 35.15 |
| Not sure | 30.28 | 32.16 | 598 | 31.23 | 56.69 |
| Sure | 69.72 | 67.84 | 1317 | 68.77 | 35.00 |
| **Social-capital** |  |  |  |  |  |
| **Perceived community participation** | | |  |  |  |
| Don't know | 53.10 | 38.17 | 873 | 45.59 | 51.32 |
| Almost none | 7.89 | 3.53 | 109 | 5.69 | 82.57 |
| Less than half | 4.52 | 9.86 | 138 | 7.21 | 47.10 |
| Half | 13.88 | 17.53 | 301 | 15.72 | 35.55 |
| More than half | 5.05 | 10.17 | 146 | 7.62 | 21.23 |
| Almost all | 15.56 | 20.75 | 348 | 18.17 | 16.95 |
| **Perceived promotion leaders** |  |  |  |  |  |
| Unsupportive/neutral | 32.81 | 13.69 | 444 | 23.19 | 61.36 |
| Supportive | 67.19 | 86.31 | 1471 | 76.81 | 35.76 |
| **Support from important people in swallowing LF drugs** | |  |  |  |  |
| Unsupportive/neutral | 41.22 | 19.29 | 578 | 30.18 | 71.28 |
| Supportive | 58.78 | 80.71 | 1337 | 69.82 | 29.02 |
| **Barriers** |  |  |  |  |  |
| **Effort to receive** |  |  |  |  |  |
| Small/no effort | 82.75 | 69.81 | 1460 | 76.24 | 42.47 |
| Moderate effort | 11.88 | 27.49 | 378 | 19.74 | 38.62 |
| Large effort | 5.36 | 2.70 | 77 | 4.02 | 44.16 |
| **Effort to swallow** |  |  |  |  |  |
| Small/no effort | 80.86 | 64.63 | 1392 | 72.69 | 41.52 |
| Moderate effort | 12.51 | 30.29 | 411 | 21.46 | 37.23 |
| Large effort | 6.63 | 5.08 | 112 | 5.85 | 61.61 |
| **Trust in LF drugs distribution team** |  |  |  |  |  |
| Trust | 87.70 | 91.39 | 1715 | 89.56 | 38.43 |
| Neutral | 10.20 | 7.47 | 169 | 8.83 | 69.23 |
| Don't trust | 2.10 | 1.14 | 31 | 1.62 | 77.42 |
| **Costs to receive** |  |  |  |  |  |
| No cost/low cost | 87.38 | 83.82 | 1639 | 85.59 | 39.11 |
| Moderate cost | 11.25 | 9.75 | 201 | 10.50 | 55.22 |
| High cost | 1.37 | 1.76 | 30 | 1.57 | 36.67 |
| **Costs to swallow** |  |  |  |  |  |
| No cost/low cost | 92.11 | 84.44 | 1690 | 88.25 | 40.00 |
| Moderate cost | 6.94 | 8.71 | 150 | 7.83 | 51.33 |
| High cost | 0.95 | 1.97 | 28 | 1.46 | 32.14 |
| **Remember receive LF drugs** |  |  |  |  |  |
| Easy | 46.79 | 48.24 | 910 | 47.52 | 29.67 |
| Neutral | 28.18 | 36.93 | 624 | 32.58 | 49.52 |
| Difficult | 25.03 | 14.83 | 381 | 19.90 | 58.01 |
